# One in Three Patients With Chronic Lateral Ankle Instability Has a Cartilage Lesion

**DOI:** 10.1177/03635465221084365

**Published:** 2022-04-06

**Authors:** Emma J. Wijnhoud, Quinten G.H. Rikken, Jari Dahmen, Inger N. Sierevelt, Sjoerd A.S. Stufkens, Gino M.M.J. Kerkhoffs

**Affiliations:** * Department of Orthopedic Surgery, Amsterdam Movement Sciences, Amsterdam UMC–Location AMC, University of Amsterdam, Amsterdam, the Netherlands; † Academic Center for Evidence-Based Sports Medicine, Amsterdam UMC, Amsterdam, the Netherlands; ‡ Amsterdam Collaboration for Health and Safety in Sports, International Olympic Committee Research Center, Amsterdam UMC, Amsterdam, the Netherlands; § Orthopedic Department, Xpert Clinics, Specialized Center of Orthopedic Research and Education, Amsterdam, the Netherlands; ‖ Orthopedic Department, Spaarnegasthuis Academy, Hoofddorp, the Netherlands; Investigation performed at the Department of Orthopaedic Surgery, Amsterdam UMC–Location AMC, Amsterdam, the Netherlands

**Keywords:** ankle, chronic lateral ankle instability, osteochondral lesion, incidence

## Abstract

**Background:**

Chronic lateral ankle instability (CLAI) is associated with the presence or development of intra-articular pathologies such as chondral or osteochondral lesions, or (O)CLs. Currently, the incidence of (O)CLs in patients with CLAI is unknown.

**Purpose:**

To determine the incidence of (O)CLs in patients with CLAI.

**Study Design:**

Systematic review and meta-analysis; Level of evidence, 4.

**Methods:**

A literature search was conducted in the PubMed (MEDLINE), Embase (Ovid), and Cochrane databases for articles published from January 2000 until December 2020. Two authors independently screened the search results and conducted the quality assessment using the methodological index for non-randomized studies (MINORS) criteria. Clinical studies were included that reported findings on the presence of ankle (O)CLs based on pre- or intraoperative diagnostic measures in patients with CLAI (>6 months of symptoms). Patient and lesion characteristics were pooled using a simplified method. Lesion characteristics included localization and chondral and osteochondral involvement. The primary outcome was the incidence of (O)CLs in ankles with CLAI. A random-effects model with 95% CIs was used to analyze the primary outcome. The distribution of (O)CLs in the ankle joint was reported according to talar or tibial involvement, with medial and lateral divisions for talar involvement.

**Results:**

Twelve studies were included with 2145 patients and 2170 ankles with CLAI. The pooled incidence of (O)CLs in ankles with CLAI was 32.2% (95% CI, 22.7%-41.7%). Among all lesions, 43% were chondral and 57% were osteochondral. Among all (O)CLs, 85% were located on the talus and 17% on the distal tibia. Of the talar (O)CLs, 68% were located medially and 32% laterally.

**Conclusion:**

(O)CLs were found in up to 32% of ankles with CLAI. The most common location was the talus (85%). Furthermore, most lesions were located on the medial talar dome (68%). These findings will aid physicians in the early recognition and treatment of ankle (O)CLs in the context of CLAI.

Lateral ankle sprains are a common injury.^15^ By and large, the mechanism of injury is an inversion sprain, in which the lateral ankle ligament complex is most often affected.^15,42^ As a consequence, up to 40% of patients may develop chronic lateral ankle instability (CLAI) despite appropriate treatment of the initial sprain.^9^ Patients with CLAI have persistent complaints of pain, recurrent ankle sprains and instability, swelling, and decreased function.^7^ Repeated trauma or microtrauma to the tibiotalar joint during recurrent ankle sprains may predispose the ankle to or increase the development of intra-articular pathology, such as a chondral lesion or osteochondral lesion, referred to collectively as or (O)CL.^5,18,28,32,38^ (O)CLs are characterized by damage to the articular cartilage and may be accompanied by a compromised subchondral bone layer.^34^ These lesions are particularly problematic, as symptoms of CLAI may be worsened by (O)CLs and in turn increase their development.^13,21,22^ As a result, concomitant (O)CLs of the ankle may accelerate whole joint degeneration and development of osteoarthritis in the context of CLAI.^11,25,39-41^ The incidence of (O)CLs in patients with CLAI is unknown, however. Understanding the incidence of (O)CLs in patients with CLAI will aid physicians in the early recognition and treatment of ankle (O)CLs and may prevent the development of lesion size and cysts, hinder the deterioration of subchondral bone, and improve clinical outcomes. Furthermore, a high incidence of (O)CLs in patients with CLAI may indicate the need for additional diagnostic measures at an earlier stage and the use of adjuvant arthroscopy for patients with CLAI undergoing surgery to address their complaints. The primary aim of the present study is therefore to determine the incidence of (O)CLs in patients with CLAI. The secondary aim is to determine the distribution of these (O)CLs in the ankle joint according to talar or tibial involvement and to determine the medial or lateral localization of talar lesions.

## Methods

This study was developed using the framework outlined in the guidelines provided by the PRISMA (Preferred Reporting Items for Systematic Reviews and Meta-Analyses) statement.^24^ The study protocol was prospectively registered in the PROSPERO registry for systematic reviews (CRD42021233410).

### Search Strategy

A systematic literature search was conducted in the PubMed (MEDLINE), Embase (Ovid), and Cochrane databases for articles published from January 2000 until December 2020. Backward citation chaining was applied to identify additional eligible records. The full literature search is provided in Appendix Table A1 (available in the online version of this article).

### Eligibility Criteria and Study Selection

Clinical studies were included that reported findings on (O)CLs of the ankle based on pre- or intraoperative diagnostic measures in patients with CLAI. The exclusion criteria are displayed in Table 1. Two authors (E.J.W. and Q.G.H.R.) independently examined potentially relevant articles, using Rayyan.^31^ Titles and abstracts and subsequent full-text articles were screened for eligibility and discussed by the 2 authors in case of disagreement. When a conflict of study eligibility remained, the judgment of the senior author (G.M.M.J.K.) was decisive. Authors were contacted by email in case data were incomplete or separate data were required for inclusion. If no response was recorded, 2 reminder emails were sent, after which a study was excluded if no response was received.

**Table 1:** Exclusion Criteria

Lateral ankle instability <6 months
No time reported on symptom duration, postinjury duration, and time after which nonoperative treatment had failed in patients with chronic lateral ankle instability
Published before 2000
Full text not available
No separate data available
Patient cohort overlap in different studies; studies excluded with the lowest number of patients

### Quality Assessment of Studies

The methodological index for non-randomized studies (MINORS) was utilized to assess the methodological quality of the studies.^35^ Studies were independently graded by 2 authors (E.J.W. and Q.G.H.R.). In case of disagreement on the MINORS score for individual studies, the judgment of the senior author (G.M.M.J.K.) was decisive.

### Terminology

For the purpose of this study, CLAI was defined as a minimum 6 months of symptom duration, postinjury duration, or time being unresponsive to nonoperative treatment.^**^ Symptoms of CLAI included, but were not limited to, recurrent ankle sprains, persistent feeling of ankle instability, ankle pain, ankle swelling, or decreased function.^7^ The postinjury duration is defined as the period from initial injury to examination or surgery.

Chondral and osteochondral lesions were analyzed altogether as (O)CLs for the primary and secondary outcomes in the present study. Where possible, lesions were subcategorized according to chondral or osteochondral involvement of the lesion. Chondral damage included chondral lesions, chondral flaps, grade 1 to 3 of the Outerbridge staging system,^30^ and grade 1 to 2 of the staging system compiled by Sugimoto et al.^37^ Osteochondral damage included osteochondral lesions, full-thickness lesions, grade 4 of the Outerbridge staging system,^30^ grade 3 of the staging system compiled by Sugimoto et al^37^ and stage 1 to 4 of the Berndt and Harty^2^ classification. In addition, a distinction was made between primary and secondary (O)CLs. (O)CLs were classified as primary lesions if patients had not undergone previous surgery for them. Lesions that failed surgical treatment were classified as secondary lesions.

### Data Extraction

A predesigned extraction form was used and piloted before data extraction. Data were extracted from each study and cross-verified by 2 independent investigators (E.J.W. and Q.G.H.R.). Study characteristics and baseline patient and lesion characteristics were extracted from the selected studies: year of publication, study design, number of patients with CLAI, number of ankles with CLAI, affected laterality, sex, age, body mass index, symptom duration, postinjury duration, number of ankles with an osteophyte, method of (O)CL diagnosis, number of ankles with an (O)CL, total number of (O)CLs, type of (O)CL, and (O)CL location. The method of (O)CL diagnosis consisted of any measure that identified (O)CLs pre- or intraoperatively. When data were available on (O)CL characteristics detected by preoperative imaging and intraoperative measures, the lesion characteristics from intraoperative measures were considered leading where applicable (eg, lesion location). (O)CL distribution was extracted according to talar or tibial involvement. When there was involvement of the talus and tibia in the same ankle, a lesion was classified as bipolar.^33^ If available, the anatomic locations of talar and tibial lesions were extracted. The anatomic locations of talar lesions were divided into medial and lateral for the purposes of this study.

### Statistical and Data Analysis

Baseline patient and lesion characteristics were pooled and weighted according to the number of patients and ankles per study, respectively. Incidence rates of (O)CLs in ankles with CLAI were pooled by means of a random-effects model with inverse variance weighting. Owing to variability in effect sizes among studies, a random-effects model was used.^4^ Between-study heterogeneity was assessed using the *I*^2^ statistic. Heterogeneity was considered low when *I*^2^ values were <25%, moderate when 25% to 50%, and high when >50%.^12^ A sensitivity analysis was performed to assess the influence of one outlier study (Sugimoto et al^38^) on the incidence of (O)CLs in ankles with CLAI. Secondary outcomes were analyzed using a simplified pooling technique where possible. The distribution of talar and tibial lesions in the ankle joint, as well as the localization of lesions on the talus (ie, medial or lateral), was calculated by dividing the number of (O)CLs by the total number of (O)CLs available in the studies reporting on specific locations—for example, the proportion of tibial (O)CLs in the total number of ankle (O)CLs. Therefore, the combined distribution of lesions may not add to 100%, as a number of studies cited various locations.^26^ If a study did not indicate the amount of (O)CLs per ankle joint, the total number of (O)CLs was calculated with 1 lesion per ankle. Data analysis was conducted using Stata Version 15 (StataCorp LP) and R Version 3.2.3 (R Foundation for Statistical Computing) using the metafor package.^45^

## Results

### Search Results

**Figure 1.**
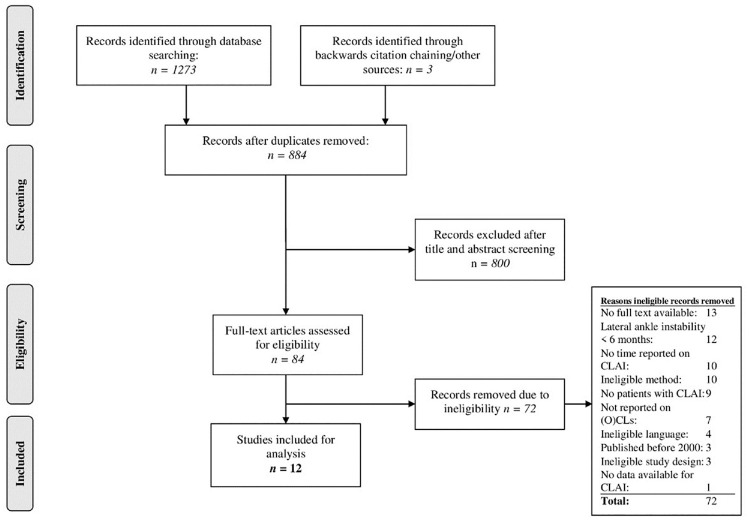
PRISMA (Preferred Reporting Items for Systematic Reviews and Meta-Analyses) flow diagram of study selection. CLAI, chronic lateral ankle instability; (O)CL, (osteo)chondral lesion.

The literature search yielded 1273 records, of which 12 studies were included for analysis (Figure 1): 3 prospective^20,27,38^ and 9 retrospective.^5,8,17-19,23,28,32,46^

### Methodological Quality

With regard to the MINORS score, consensus was reached between the reviewers in all studies. Six noncomparative studies^5,8,17,27,28,46^ had an average score of 8.8 out of 16 points (range, 7-11). Six comparative studies^18-20,23,32,38^ scored an average of 17 out of 24 points (range, 15-19). The MINORS score per study is provided in the Appendix Table A2 (available online).

### Patient and Lesion Characteristics

In total, 2170 ankles with CLAI were included (2145 patients). An overview of the pooled characteristics and per individual study is available in Table 2. CLAI was predominantly defined as a combination of complaints (eg, repetitive spraining, failure of nonoperative treatment), physical examination (eg, positive anterior drawer or talar tilt test), and stress radiography.^††^ Definitions for CLAI per study are reported in Appendix Table A3 (available online).

**Table 2:** Study and Patient Characteristics^*a*^

			CLAI, No.		No. (%)		Mean (Range)		Duration, mo, Mean (Range)		
First Author	Year	Study Design	Patients	Ankles	Right Ankle Involved	Male Sex	Age, y	BMI	Symptom	Postinjury^*b*^	Osteophytes, %
Choi^5^	2008	Retrospective case series	64	65	39 (60.0)	44 (68.8)	27.0 (15-57)			19 (6-84)	10.8
DiGiovanni^8^	2000	Retrospective review	61	61		32 (52.5)	35.0 (17-59)			18 (7-72)	
Hou^17^	2020	Retrospective study	220	220		164 (74.5)	33.9	24.8		33.2 (≥6)	13.2
Hua^18^	2010	Retrospective case series	85	87		58 (68.2)	24.4 (14-36)			29.2 (7-96)	26.4
Kim^19^	2010	Retrospective comparative study	69	74		49 (66.2)^*c*^	27.6 (15-55)		23.8		0.0
Ko^20^	2020	Randomized controlled trial	43	43	16 (37.2)	34 (79.1)	29.5	25.8	89.4		
Li^23,*d*^	2017	Retrospective study	89	89						— (>6)	
Nery^28^	2011	Retrospective case series	38	38	20 (52.6)	24 (63.2)	28.8 (15-53)			9 (6-19)	
Nery^27^	2018	Prospective study	26	26		20 (76.9)	41.4 (19-60)				
Park^32^	2016	Retrospective cohort study	188	199		136 (72.3)	29.1	24.4	26.9	26.9 (12-192)	6.5
Sugimoto^38^	2009	Prospective case series	93	99		40 (43.0)	28.7 (15-59)	22.7 (17.2-31.3)	73.6 (6-360)	74 (6-360)	
Wang^46^	2020	Retrospective case-control study	1169	1169	693 (59.3)	776 (66.4)	30.0	24.9		50	28.6
Total											
No. (%)			2145	2170	768 (58.4)	1377 (67.0)	2056	1730	415	1918	1814
Mean (range)							30.1 (24.4-41.4)	24.7 (21.8-26.0)	44.0 (23.6-103.7)	43.2 (9-74)	22.4 (20.5-24.4)^*e*^

a Some studies^19,20,32,38^ reported multiple groups. For the purpose of this study, the outcomes were pooled according to the number of ankles per group. Blank cells indicate *not available*. BMI, body mass index; CLAI, chronic lateral ankle instability.

b Hou et al^17^ defined postinjury duration as the period from initial injury to examination. All other studies defined postinjury duration as the period from initial injury to surgery.

c Kim et al^19^ was the only study from which the percentage was calculated out of the number of ankles with CLAI, instead of the number of patients with CLAI.

d The primary outcome was obtained by contacting the authors; therefore, no additional patient characteristics were available.

e Data in parentheses indicate 95% CI.

Of the 12 studies, 768 ankles were diagnosed with an (O)CL (Table 3). Of the 1162 (O)CLs, 42.6% were chondral and 57.4% osteochondral. With regard to the methods of diagnosis, all studies utilized intraoperative measures as their gold standard for diagnosis. When intraoperative measures were used, 11 studies^‡‡^ utilized ankle arthroscopy, and 1 study^8^ used arthrotomy of the ankle joint.

**Table 3:** (O)CL Incidence and Lesion Characteristics^*a*^

		Ankles With, No.			Lesions, No. (%)				
First Author	(O)CL Diagnostic Method	CLAI	(O)CL	Total (O)CLs	Primary	Secondary	Chondral	Osteochondral	(O)CL Incidence in Ankles With CLAI, %
Choi^5^	MRI and intraoperative	65	15				0	15	23.1
DiGiovanni^8^	MRI and intraoperative	61	14				12	2	23.0
Hou^17^	Intraoperative	220	98		98	0	0	98	44.5
Hua^18^	MRI and intraoperative	87	33		33	0	33	0	37.9
Kim^19^	Intraoperative	74	16				0	16	21.6
Ko^20^	Intraoperative	43	8		8	0	0	8	18.6
Li^23^	MRI and intraoperative	89	27		27	0	0	27	30.3
Nery^28^	MRI and intraoperative	38	10		10	0	0	10	26.3
Nery^27^	MRI and intraoperative	26	8				0	8	30.8
Park^32^	MRI and intraoperative	199	27		27	0	0	27	13.6
Sugimoto^38^	Intraoperative	99	76	159			137	22	76.8
Wang^46^	Intraoperative	1169	436	747			313	434	37.3
Total		2170	768	906	203 (100)	0	495 (42.6)	667 (57.4)	32.2

a Data were not uniformly reported; percentages were therefore calculated with available data. Blank cells indicate *not available*. CLAI, chronic lateral ankle instability; MRI, magnetic resonance imaging; (O)CL, (osteo)chondral lesion.

### (O)CL Incidence

**Figure 2.**
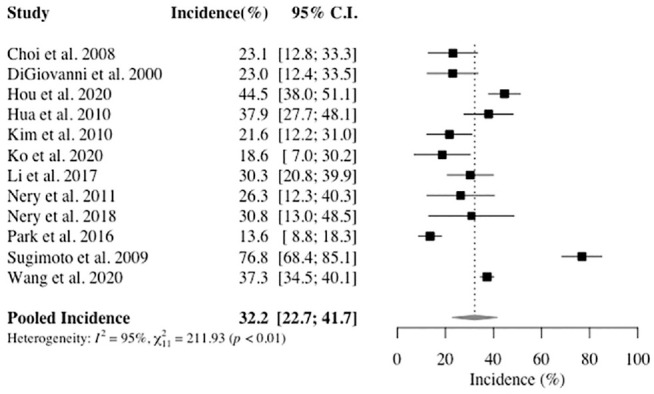
Forest plot of the incidence of (osteo)chondral lesions in ankles with chronic lateral ankle instability among the included studies. Proportions are shown with 95% CIs.

The pooled incidence of (O)CLs in ankles with CLAI was 32.2% (95% CI, 22.7%-41.7%) (Figure 2). From the primary analysis, the Sugimoto et al^38^ study was identified as a potential outlier. Hence, a sensitivity analysis was performed to examine the effect of excluding Sugimoto et al^38^ on the pooled incidence. This resulted in an (O)CL incidence of 28.0% (95% CI, 20.7%-35.4%) in ankles with CLAI (Appendix Figure A1, available online).

### Distribution of (O)CLs

**Figure 3.**
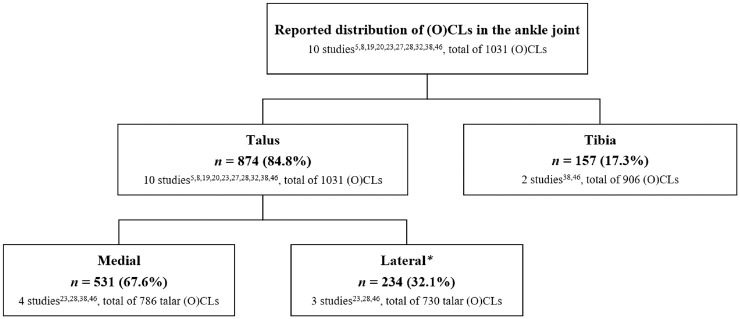
Distribution of (O)CLs in the ankle joint. Note that not all studies reported on the distribution of (O)CLs. The number of studies reporting on a specific location and the total number of (O)CLs are presented. Absolute numbers were calculated with available data and therefore do not add to 100%. *Sugimoto et al^38^ reported on “central and lateral areas” as a combined term. For that reason, these (O)CLs are excluded from the “lateral” category. (O)CL, (osteo)chondral lesion.

Ten studies^§§^ with 1031 (O)CLs reported on the distribution of (O)CLs in the ankle joint (Figure 3). An overall 84.8% of (O)CLs were talar lesions. Of the 2 studies^38,46^ that examined tibial lesions, 17.3% of (O)CLs were on the distal tibia.

For the localization of talar lesions, 67.6% were located medially, as noted in 4 studies,^23,28,38,46^ and 32.1% were located laterally, as reported in 3 studies.^23,28,46^

Two studies^38,46^ examined bipolar lesions. In total, 55 (10.7%) ankles had a bipolar lesion out of 512 ankles with an (O)CL.

## Discussion

The most important finding of the present study is that (O)CLs are present in 32% of patients with CLAI. The most common location of (O)CLs in patients with CLAI is the talus. Talar lesions were predominantly located medially. These findings highlight the importance for awareness among clinicians in the clinical workup of patients with CLAI as well as appropriate surgical preparation and management.

### (O)CL Incidence in CLAI and Clinical Relevance

Of the patients who sustain an ankle sprain, up to 40% may develop chronic ankle instability despite appropriate initial treatment.^9^ In the present study, a high proportion of patients with CLAI had an ankle (O)CL. A number of explanations for this high incidence can be considered. First, the initial ankle trauma or repeated spraining in the context of CLAI can cause the formation of an (O)CL. This is in line with the current understanding that (O)CLs predominantly occur after trauma, such as an ankle sprain or fracture.^34^ When the pooled incidence of (O)CLs in this study was compared with that in a systematic review of (O)CLs with acute ankle fractures, a similarly high incidence (45%) was noted.^26^ A second reason for cartilage damage and (O)CL development in ankles with CLAI is the “scraping” of anterior tibial osteophytes on the articular talar cartilage when the ankle is in dorsiflexion. Osteophytes are thought to provide stability as a pathophysiological response to instability or microinstability and are frequently observed in patients with CLAI.^5,17,18,32,46^ A third reason for (O)CL development in the context of CLAI is prolonged microinstability. This may lead to a suboptimal loading pattern of the ankle, which strains the articular tibiotalar cartilage and increases cartilage wear over time.^3,7,33^ The gradual cartilage degradation initiates progression of the cascade from cartilage lesion toward end-stage osteoarthritis.^6^ Chronic ankle instability can therefore be regarded as an important prognostic factor for ankle osteoarthritis and may increase the likeliness of (O)CL formation as well as the acceleration of (O)CL progression. Early detection of ankle (O)CLs in patients first presenting with CLAI is therefore crucial to limit cartilage degeneration and increase the likelihood of successful first-line nonoperative and/or operative (O)CL treatment in (minimally) symptomatic cases. Early detection is possible with the use of advanced imaging techniques such as magnetic resonance imaging (MRI) and computed tomography (CT) scanning. MRI is known to be a sensitive tool for diagnosing a talar (O)CL and is frequently used to assess the lateral ligament complex in patients with suspected CLAI.^44^ Although MRI has good sensitivity for diagnosing an (O)CL, it can overestimate lesion size when bone marrow edema is present.^47^ CT scanning allows for accurate assessment of the bony morphology of the lesion and an adequate representation of lesion size, and could therefore be considered a good alternative to MRI. The use of such a dynamic approach with MRI and/or CT may allow for the integration of a complete assessment of the morphological lesion characteristics into the individualized patient (surgical) treatment plan.^34^

Although MRI and CT provide a high degree of diagnostic accuracy, arthroscopic assessment can also be an accurate diagnostic assessment method for diagnosing an (O)CL.^44^ For patients with CLAI, the present study found a high rate of chondral lesions, which may be more challenging to assess on a diagnostic and morphological level using imaging modalities alone. Adjuvant ankle (nano)arthroscopy could be a combinatory diagnostic and therapeutic tool for patients with CLAI, especially in patients who will undergo a lateral ligament repair. The advantages of arthroscopic assessment are the direct visualization of the (O)CL, the evaluation of any concomitant intra-articular pathologies, and the ability to simultaneously address these pathologies intraoperatively. The continued developments in arthroscopic technologies, more recently by the introduction of safe and effective 2-mm arthroscopes, could provide physicians with additional tools to adequately diagnose and treat these lesions in patients with CLAI.^36^

### Lesion Distribution and Talar Localization

When the distribution of (O)CLs in the ankle joint was assessed, it was shown that lesions in patients with CLAI were primarily located on the talar dome (85%). This is in line with the findings of Martijn et al,^26^ who found that (O)CLs were most often located on the talus in patients with ankle fractures. When the localization of talar (O)CLs in patients with CLAI was evaluated, it was observed that a high rate of lesions was located medially. Lesion localization may be explained by lesion etiology. First, traumatic lesions after an ankle sprain can cause acute medial lesions and shallow chondral lateral lesions attributed to the talar dome impacting the tibial plafond and distal fibula, respectively. Second, because of the altered tibiotalar joint biomechanics in lateral ligament deficiency, gradual and diffuse lesions can occur medially and laterally. Wang et al^46^ found a postinjury duration >5 years to be associated with the presence of (O)CLs on the medial talus and hypothesized that, because of the ligament deficiency, increased talar anterior translation and internal rotation may cause peak strain on the articular cartilage. For lateral lesions, the previously mentioned scraping of anterior tibial osteophytes observed in patients with CLAI may explain the lateral localization of degenerative (O)CLs on the talus. Thus, the lesions assessed in this study may be attributable to differing causes in the acute and chronic (ie, degenerative) phases of (O)CL development. The available data on the localization of talar lesions and their possible chondral or osteochondral nature did not allow for further analysis or conclusions to be drawn, given the underreporting of data. The finding of the present study that the majority of the talar lesions were located medially is in line with that of a recent systematic review by van Diepen et al.^43^ In that study, 73% of talar lesions were located on the medial talus, but the authors did not assess the presence of lateral ankle instability.

Tibial lesions are thought to be less prevalent than talar lesions.^10^ The incidence of tibial (O)CLs in this study is comparable with that observed in the systematic review by Martijn et al^26^ (each 17%). The lower incidence of tibial (O)CLs can be hypothesized to be due to the stiffer tibial cartilage compared with talar cartilage.^1^ Caution is warranted with assessing the incidence of tibial lesions in the current review, as only 2 studies investigated the prevalence of tibial lesions.^38,46^ Further research should focus on accurately reporting concomitant (O)CLs with CLAI, their distribution and localization, and the lesion characteristics, as well as other relevant intra-articular pathologies.

### Strengths and Limitations

This is the first study to pool the incidence of (O)CLs in ankles with CLAI. A strength of this study was the use of strict inclusion and exclusion criteria. Specifically, articles were included if pre- or intraoperative assessment of (O)CLs was performed and the assessment protocol was reported in the Methods section. Moreover, almost all authors assessed the presence of an (O)CL by arthroscopy, which may mitigate the variability between diagnostic modalities (ie, imaging versus intraoperatively).

This study is not without its limitations, however. First, the level of evidence of the studies was moderate, as exemplified by the MINORS score. Second, a number of studies did not assess the incidence of (O)CLs as their primary outcome but rather reported these as concomitant injuries during surgical procedures for CLAI. It may therefore be that the pooled incidence of ankle (O)CLs in the present study underestimates the true incidence of (O)CLs, as exemplified by the high incidence found by Sugimoto et al,^38^ who utilized a rigorous 5-point intraoperative arthroscopic protocol. The varying intraoperative assessment methods throughout the studies may also explain the degree of heterogeneity among studies. Another limitation was the heterogeneous definition of CLAI in the studies. Although minimum symptom duration was defined as 6 months in the present study, the different criteria could lead to the inclusion of a heterogeneous study sample. This makes it difficult to compare results across studies and suggests that consensus has not been reached on the definition of CLAI in the literature. Another important limitation of the present study was that it was not known from the included studies if lesions were symptomatic (O)CLs. A large portion of lesions were chondral in nature, and it might be that these lesions would not develop into a symptomatic lesion. The results of this study should be interpreted with caution and in the context of the study design.

## Conclusion

In 1 of 3 patients with CLAI, an (O)CL is present. In this study, the most common location of (O)CLs in patients with CLAI was the talar dome (85%). Talar lesions were predominantly located medially (68%). These findings highlight the importance for awareness among clinicians in the clinical workup of patients with CLAI as well as appropriate surgical preparation and management.

## Supplemental Material

sj-pdf-1-ajs-10.1177_03635465221084365 – Supplemental material for One in Three Patients With Chronic Lateral Ankle Instability Has a Cartilage Lesion Click here for additional data file. Supplemental material, sj-pdf-1-ajs-10.1177_03635465221084365 for One in Three Patients With Chronic Lateral Ankle Instability Has a Cartilage Lesion by Emma J. Wijnhoud, Quinten G.H. Rikken, Jari Dahmen, Inger N. Sierevelt, Sjoerd A.S. Stufkens and Gino M.M.J. Kerkhoffs in The American Journal of Sports Medicine

## Footnotes

** References 5, 14, 16, 17, 19, 20, 27-29, 32, 46

†† References 5, 8, 17-20, 27, 28, 32, 38, 46

‡‡ References 5, 17-23, 23, 27, 28, 32, 38, 46

§§ References 5, 8, 19, 20, 23, 27, 28, 32, 38, 46
