# Supplementary material for: One in Three Patients With Chronic Lateral Ankle Instability Has a Cartilage Lesion
Source: Am J Sports Med. 2022 Apr 6;51(7):1943–51. doi: 10.1177/03635465221084365 (PMC10240649; doi:10.1177/03635465221084365)
Supplement: sj-pdf-1-ajs-10.1177_03635465221084365 – Supplemental material for One in Three Patients With Chronic Lateral Ankle Instability Has a Cartilage Lesion [file sj-pdf-1-ajs-10.1177_03635465221084365.pdf]

Incidence of Cartilage Lesions in Patients with Chronic Lateral Ankle Instability

Appendix

Table A1: The full literature search

Databases		
PubMed (MEDLINE), EMBASE (Ovid), Cochrane Library	Before deduplicating	After deduplicating
Total	1273	**881**

Full electronic search strategy used in this systematic review.

PubMed (MEDLINE)

#	Searches	Results
1	"Osteochondritis Dissecans"[MeSH]	1628
2	osteochondritis dissecans[tiab] OR osteochondrosis dissecans[tiab] OR osteochondrolysis[tiab] OR OCD[tiab] OR OLT[tiab]	16081
3	(osteochondral[tiab] OR chondral[tiab] OR transchondral[tiab] OR cartilage*[tiab]) AND (defect*[tiab] OR lesion*[tiab] OR damag*[tiab] OR injur*[tiab])	24731
4	#1 OR #2 OR #3	30411
5	("Ankle Joint"[Mesh] OR "Ankle"[Mesh] OR "Talus"[Mesh] OR "Lateral Ligament, Ankle"[Mesh] OR "Ankle Fractures"[Mesh] OR "Ankle Injuries"[Mesh] OR "Ligaments"[Mesh]) AND "Joint Instability"[Mesh]	7204
6	(talus[tiab] OR talar*[tiab] OR ankle*[tiab]) AND (instab*[tiab] OR unstab*[tiab] OR laxit*[tiab] OR hypermobilit*[tiab] OR stable[tiab] OR stabilit*[tiab])	7394
7	CLAI[tiab]	517
8	#5 OR #6 OR #7	13126
9	#4 AND #8	**600**

EMBASE (Ovid)

#	Searches	Results
1	osteochondritis dissecans/	3076
2	(osteochondritis dissecans or osteochondrosis dissecans or osteochondrolysis or OCD or OLT).ti,ab,kw.	24836
3	((osteochondral or chondral or osteochondral or transchondral or cartilage*) and (defect* or lesion* or damag* or injur*)).ti,ab,kw.	44485
4	1 or 2 or 3	68566
5	ankle instability/	2147
6	(talus/ or exp ankle/ or exp ankle lateral ligament/ or exp ankle injury/ or exp ankle fracture/ or joint ligament/ or ligament/) and (exp joint instability/ or joint laxity/)	2629
7	((talus or talar* or ankle*) and (instab* or unstab* or laxit* or hypermobilit* or stable or stabilit*)).ti,ab,kw.	9964
8	CLAI.ti,ab,kw.	523
9	5 or 6 or 7 or 8	12484
10	4 and 9	**587**

Cochrane Library

#	Searches	Results
1	MeSH descriptor: [Osteochondritis] explode all trees	31
2	(osteochondritis dissecans OR osteochondrosis dissecans OR osteochondrolysis OR OCD OR OLT):ti,ab,kw (Word variations have been searched)	2090
3	((osteochondral OR chondral OR transchondral OR cartilage*) AND (defect* or lesion* or damag* or injur*)):ti,ab,kw	1414
4	#1 or #2 or #3	3465
5	MeSH descriptor: [Joint Instability] explode all trees	741
6	MeSH descriptor: [Talus] explode all trees	34
7	MeSH descriptor: [Ankle] explode all trees	499
8	MeSH descriptor: [Ankle Joint] explode all trees	698
9	MeSH descriptor: [Ankle Injuries] explode all trees	708
10	MeSH descriptor: [Ankle Fractures] explode all trees	150
11	MeSH descriptor: [Lateral Ligament, Ankle] explode all trees	27
12	MeSH descriptor: [Ligaments] explode all trees	1142
13	((talus OR talar* OR ankle*) AND (instab* or unstab* or laxit* or hypermobilit* or stable or stabilit*)):ti,ab,kw (Word variations have been searched)	1326
14	(CLAI):ti,ab,kw	4
15	#5 or #6 or #7 or #8 or #9 or #10 or #11 or #12 or #13 or #14	4146
16	#4 and #15	**86**

Table A2: MINORS score per included study

Study	A clearly stated aim	Inclusion of consecutive patients	Prospective collection of data	Endpoint appropiate to the aim of the study	Unbiased assesment of the study endpoint	Follow up period appropiate to the aim of the study	Lost of follow up less than 5%	Prospective calculation of the study size	Additional criteria comparative research				Total
									An adequate control group	Contemporary groups	Baseline equivalence of groups	Adequate statistical analyses	
Choi et al.[5]	1	2	2	1	1	2	2	0	-	-	-	-	11/16
DiGiovanni et al.[8]	2	2	0	2	1	0	0	0	-	-	-	-	7/16
Hou et al.[17]	1	2	0	2	2	0	2	0	-	-	-	-	9/16
Hua et al.[18]	1	2	2	2	1	2	1	0	2	2	1	2	18/24
Kim et al.[19]	0	2	2	1	1	2	0	0	2	2	2	2	16/24
Ko et al.[20]	1	2	2	1	1	2	1	2	2	2	2	1	19/24
Li et al.[23]	1	2	0	1	1	2	0	0	2	2	2	2	15/24
Nery et al.[28]	1	2	0	1	1	2	1	0	-	-	-	-	8/16
Nery et al.[27]	1	2	2	1	1	2	0	0	-	-	-	-	9/16
Park et al.[32]	1	2	2	1	1	2	1	0	2	2	2	2	18/24
Sugimoto et al.[38]	2	2	0	2	1	2	0	0	2	2	1	2	16/24
Wang et al.[46]	2	2	0	2	1	2	0	0	-	-	-	-	9/16

Table A3: Specific inclusion criteria studies have used to define CLAI

Author	Ankle Instability Definition	Inclusion criteria used to select patients with CLAI	
		**History signs**	**Physical examination and additional diagnostic signs**
Choi et al.[5]	Chronic Lateral Ankle Instability	1. Repetitive ankle sprain injury*.<br>2. Failure of non-operative treatments for 6 months.	1) Instability with pain > grade 2 on an anterior drawer test*.<br>2) ≥ 10° difference on talar tilt test in comparison with the contralateral side.<br>* 1) or 1)
DiGiovanni et al.[8]	Chronic Lateral Ankle Instability	1. Indication of CLAI by history.<br>2. Failure of non-operative treatments.	1. Indication of CLAI by physical examination and, if needed, stress radiographs.
Hou et al.[17]	Chronic Mechanical Ankle Instability	1. History of ≥ 1 serious ankle sprain.<br>2. Injury-examination duration > 6 months.<br>3. Recurrent sprain (defined as > 2 sprains in 6 months), and/or experiencing feelings of instability.	1. Grade III ligament lesion confirmed by both MRI, a positive anterior drawer test and a positive talar tilt test.
Hua et al.[18]	Chronic Ankle Instability	1. Intra-articular symptoms.<br>2. Failure of non-operative treatments.	Not specified.
Kim et al.[19]	Chronic Lateral Ankle Instability	1. Symptoms for ≥ 6 months.<br>2. Failure of non-operative treatments.	Not specified.
Ko et al.[20]	Chronic Lateral Ankle Instability	1. Symptoms (recurrent sprain or a feeling of giving way) for ≥ 6 months.	1. MRI findings of ATFL abnormality.<br>2. ≥2 of the following findings: anterior drawer test finding > 10 mm; anterior talar translation > 10 mm on anterior drawer stress radiograph; talar tilt angle > 10° on varus stress radiograph.
Li et al.[23]	Chronic Lateral Ankle Instability	Not specified.	1. Positive anterior drawer test, compared contralaterally.
Nery et al.[28]	Chronic Lateral Ankle Instability	1. > 2 episodes of functional instability (feeling of giving way) of the ankle.<br>2. Failure of non-operative treatments for ≥ 6 months.	1. > Grade 2 mechanical laxity on the clinical and radiographic anterior drawer test.
Nery et al.[27]	Chronic Lateral Ankle Instability	1. Pain, giving way, and recurrent instability of the resulting from an injury to the lateral ligament complex.<br>2. Failure of non-operative treatments for ≥ 6 months.	1. Positive anterior drawer test.
Park et al.[32]	Chronic Lateral Ankle Instability	1. Recurrent giving way or a feeling of apprehension with pain.<br>2. Failure of non-operative treatments for ≥ 6 months.	1. Positive findings on stress radiography.
Sugimoto et al.[38]	Recurrent or Chronic Lateral Ankle Instability	1. Recurrent giving-way or a feeling of apprehension with pain.<br>2. Inversion sprains in the past.<br>3. Failure of non-operative measures for ≥ 3 months.	1. Findings on physical examination.<br>2. Findings on stress radiography (talar tilt test or anterior drawer test).
Wang et al.[46]	Chronic Lateral Ankle Instability	1. History of ≥ 1 significant ankle sprain.<br>2. Ankle instability persisting > 6 months.<br>3. Feeling of the previous injured ankle joint 'giving way' and/or recurrent sprain (> 2 sprains in 6 months) and/or 'feelings of instability'	1. Injury of the lateral ligaments (ATFL and/or CFL)

Figure A1: Sensitivity analysis

Study	Incidence(%)	95% C.I.
Choi et al. 2008	23.1	[12.8; 33.3]
DiGiovanni et al. 2000	23.0	[12.4; 33.5]
Hou et al. 2020	44.5	[38.0; 51.1]
Hua et al. 2010	37.9	[27.7; 48.1]
Kim et al. 2010	21.6	[12.2; 31.0]
Ko et al. 2020	18.6	[7.0; 30.2]
Li et al. 2017	30.3	[20.8; 39.9]
Nery et al. 2011	26.3	[12.3; 40.3]
Nery et al. 2018	30.8	[13.0; 48.5]
Park et al. 2016	13.6	[8.8; 18.3]
Wang et al. 2020	37.3	[34.5; 40.1]
**Pooled Incidence**	**28.0**	**[20.7; 35.4]**

Heterogeneity: $I^2 = 90\%$, $\chi^2_{10} = 102.65$ ($p < 0.01$)

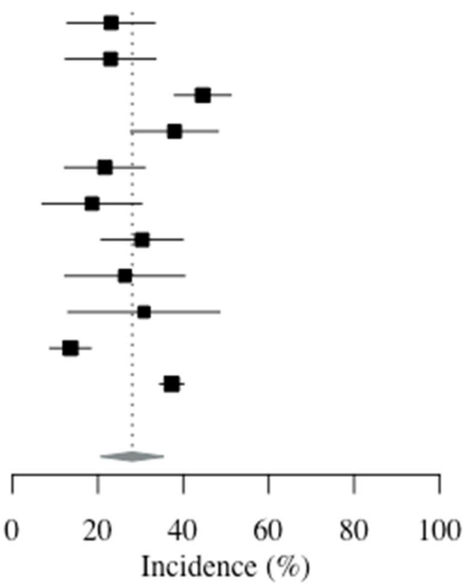

(O)CL incidence in ankles with CLAI without the study of Sugimoto et al.[38]
